# SUR7 deletion in *Candida albican*s impacts extracellular vesicle features and delivery of virulence factors

**DOI:** 10.1002/jex2.82

**Published:** 2023-05-02

**Authors:** James A. McKenna, Donovan Garcia‐Ceron, Mark R. Bleackley, Long Yu, Vincent Bulone, Marilyn A. Anderson

**Affiliations:** ^1^ Department of Biochemistry and Chemistry La Trobe Institute for Molecular Science, La Trobe University VIC Australia; ^2^ School of Agriculture Food and Wine The University of Adelaide Waite Campus SA Australia; ^3^ Centre for Marine Bioproducts Development, College of Medicine & Public Health Flinders University SA Australia; ^4^ Division of Glycoscience Department of Chemistry, School of Engineering Sciences in Chemistry, Biotechnology and Health, Royal Institute of Technology (KTH) AlbaNova University Centre Stockholm Sweden

**Keywords:** *Candida albicans*, extracellular vesicles, Sur7, virulence

## Abstract

Extracellular vesicles (EVs) from human fungal pathogens have been implicated in fungal virulence, yet little is known about their role in the host‐pathogen interaction. Progress has been hampered by the lack of a specific marker for fungal EVs that can be used to monitor EV isolation and tracking in biological systems. Here we report the effect of a *SUR7* gene knockout on the production, properties, and role of EVs in the virulence of *Candida albicans*. Sur7 is a component of the membrane compartment of Can1 (MCC) complex and is enriched in the EVs from *C. albicans* and other fungal species. MCC is a plasma membrane complex which together with the eisosome, a cytoplasmic protein complex, is a key regulator in plasma membrane organisation and plasma membrane associated processes. The *SUR7* knockout strain produces smaller EVs than the wild‐type (WT) with different protein and carbohydrate cargos. Furthermore, proteins with known roles in Candida pathogenesis were present in WT EVs and absent or diminished in the *sur7*Δ EVs. We demonstrate that the reduced virulence of the *sur7Δ* cells can be partially restored with EVs from a WT strain. These findings demonstrate the importance of Sur7‐like proteins in the biogenesis of EVs in fungi and enhance our understanding of the role of fungal EVs in human pathogenesis.

## 1 INTRODUCTION

Fungal diseases affect over a billion people worldwide (Brown et al., 2012). While the majority of mycoses are superficial, non‐life threatening infections of the skin, nails, hair follicles and mucosal surfaces, more than 150 million people each year suffer from serious infections that severely impact the quality of their lives and can be fatal (Bongomin et al., 2017). In 2017 the fungal disease burden was estimated at more than $7.2 billion (USD) in the USA alone with more than $1.2 billion (USD) attributed to hospitalizations from *Candida* infections (Benedict et al., 2019). Several *Candida* species are commensal organisms of the skin and gut microbiota, and they are among the most common fungi that cause human disease (Pappas et al., 2018). Most *Candida* infections are mucosal, with over 3 million cases of oral or oesophageal candidiasis reported annually. In addition, 70% of women experience an episode of vulvovaginal candidiasis during their lifetime (Bongomin et al., 2017). However, the estimated 750,000 annual cases of invasive candidiasis are of the highest concern as these infections contribute most to the morbidity and mortality (Bongomin et al., 2017). Systemic candidemia infections are typically nosocomially acquired and have an associated mortality of 10%–20% (Pappas et al., 2018). Once identified, *Candida* infections can be treated with antifungal agents from the azole, echinocandin and polyene families, although multidrug resistance among *Candida* species is becoming more prevalent (Lee et al., 2021). Thus, rapid detection and identification of invasive candidiasis is required to promote improved patient outcomes.

Extracellular vesicles or EVs are defined as “particles that are naturally released from the cell, are delimited by a lipid bilayer and cannot replicate” (Théry et al., 2018). EVs provide a non‐classical method for the coordinated secretion of biological molecules. In higher eukaryotes such as humans this facilitates intercellular communication (Maas et al., 2017) with further implications in diseases such as cancers (Tai et al., 2019), cardiovascular disease (Chong et al., 2019) and neurological disorders (Osier et al., 2018). EVs can also facilitate communication between organisms and have recently been implicated in the progression of microbial infections by bacteria and fungi (Herkert et al., 2019; Joffe et al., 2016). Examples include *Staphylococcus aureus* (Tartaglia et al., 2018), *Mycobacterium tuberculosis* (Gupta & Rodriguez, 2018), *Trichophyton interdigitale* (Bitencourt et al., 2018) and *Cryptococcus gattii* (Bielska et al., 2018).

Fungal EVs have diverse biological cargos including lipids (Wolf et al., 2015), proteins (Bleackley et al., 2019; Karkowska‐Kuleta et al., 2020; Zhao et al., 2019), carbohydrates (Rodrigues et al., 2007), nucleic acids (Peres da Silva et al., 2019; Peres da Silva, Puccia et al., 2015) and other metabolites (Bleackley et al., 2020). The composition of *Candida albicans* EVs, which has only recently been partially elucidated, may reflect the composition of EVs from other fungi (Dawson et al., 2020; Konečná et al., 2019; Peres da Silva, Puccia et al., 2015; Vargas et al., 2015; Zarnowski et al., 2018).

Fungal EVs were first identified from Cryptococcus spp. and thus this system has been the best studied. *Cryptococcus neoformans* EVs deliver virulence factors to host brain tissue and are able to protect Cryptococcus cells from host‐derived reactive oxygen stress (Silva et al., 2019). Furthermore, a *Cryptococcus deuterogattii* knockout mutant that was hypovirulent in the *Galleria mellonella* infection model had its phenotype reverted to wild‐type (WT) when EVs produced by WT cells were coinfected with the knockout line, thus implicating EVs in the promotion of infection (Castelli et al., 2022). To date little has been reported about the potential role of *C. albicans* EVs in the infection process, although there is evidence that *Candida* EVs can elicit an immune response. For example, pre‐inoculation of *G. mellonella* larva with *C. albicans* EVs prior to infection with *C. albicans* decreased the number of colony forming units and increased larval survival (Vargas et al., 2020). *C. albicans* EVs have also been demonstrated to be internalized by macrophages and to elicit an immune response *in vitro* (Vargas et al., 2020). More recently the same group demonstrated that mice that had been pre‐immunized with *C. albicans* EVs survived a challenge with *C. albicans* whereas control mice that had not been preimmunised with EVs died (Vargas et al., 2020). *C. albicans* EVs have also been implicated in the enhanced resistance of biofilms against antifungal therapeutics (Zarnowski et al., 2018).

We reported the proteome of *C. albicans* EVs in an earlier publication and identified several potential EV markers based on enrichment in EVs compared to whole cell lysate (WCL) (Dawson et al., 2020). One of the most interesting potential markers was Sur7 which has similar topology to the tetraspanins that are used as markers for mammalian EVs. Sur7 is a plasma membrane protein that is a component of the membrane compartment of Can1 (MCC)/eisosome complex or site of endocytosis (Walther et al., 2006). The MCC/eisosome complex is located on the plasma membrane in stable furrows of 300 nm by 50 nm and 50 nm in depth (Lanze et al., 2020). MCC/eisosomes maintain nutrient transporters at the cell surface, such as the Can1 arginine symporter, and protect them from endocytosis and degradation. MCC/eisosomes are also involved in the stress response to membrane tension, nutrition, cell wall integrity, oxidation and copper toxicity (Lanze et al., 2020). The properties of homozygous *C. albicans* knockout strains of *SUR7* (*sur7Δ*) have been the subject of several reports (Alvarez et al., 2008; Bernardo & Lee, 2010; Douglas & Konopka, 2019; Wang et al., 2011, 2016). The phenotypes of these strains include reduced biofilm formation, defective endocytosis, sensitivity to copper, oxidation and cell wall perturbation. The *sur7Δ C. albicans* lines are also defective in macrophage killing *in vitro* and virulence *in vivo* (Alvarez et al., 2008; Bernardo & Lee, 2010; Douglas & Konopka, 2019; Douglas et al., 2011; Wang et al., 2011, 2016). Here we report the characterization of EVs from *sur7Δ C. albicans* strains and compare them to EVs from the WT strain and EVs from *sur7Δ C. albicans* after complementation with *SUR7*. We describe how differences in the EV cargo contribute to a loss of virulence of *sur7Δ C. albicans*. We also report that the phenotype of decreased virulence of the *sur7Δ C. albicans* cell line can be partially reverted by addition of EVs from the WT line.

## 2 MATERIALS AND METHODS

### 2.1 Fungal isolates and growth of fungal cultures

The *C. albicans* cell lines DIC185 (WT), YJA11 (*sur7Δ*) and YJA12 (complement or comp) were a kind gift from Professor James Konopka, Dept. Molecular Genetics and Microbiology, Stony Brook University. They were produced as described by Alvarez et al. (2008). Strains were streaked onto Yeast, Peptone, Dextrose (YPD) (1% yeast extract, 2% peptone, 2% glucose) agar prior to inoculation in liquid culture. A 5 mL culture in YPD (50 mL tube, Greiner) of each strain was grown overnight with shaking (300 rpm) at 30°C and diluted in RPMI to produce a final OD_600_ of 10–12. Each strain (5 mL) was then transferred to 200 mL of RPMI 1640 medium (2% glucose, pH 7, Sigma) (2 × 100 mL, in 200 mL baffled flasks) and incubated at 30°C for 48 h with shaking at 150 rpm. Cells were removed by centrifugation for 6 min at 3220×g. The supernatant was then passed through a sterile 0.45 μm syringe filter prior to EV isolation.

### 2.2 Isolation of EVs from *C. albicans*

EVs were isolated essentially as described by Garcia‐Ceron et al. (2021). Briefly, the supernatants from the *C. albicans* cultures were concentrated to approximately 500 μL using 15 mL 100‐kDa NMWCO centrifugal filter units (Merck). The concentrates were mixed with the fluorescent lipophilic dye FM5‐95 (Thermo Fisher) at a final concentration of 1.75 μM and incubated for 15 min in the dark. The samples were loaded onto a 10‐mL Sepharose CL 2B column (Sigma) that had been pre‐equilibrated with eight column volumes of Dulbecco's phosphate‐buffered saline (DPBS) (Gibco). For each sample, 36 fractions (approximately 280 μL each) were eluted with DPBS and collected in black microtiter plates (Greiner). Fluorescence (excitation and emission wavelengths, 560 and 734 nm, respectively) was measured from each fraction in a SpectraMax M2 plate reader (Molecular Devices). Fractions (typically 10–18) with consistent positive relative fluorescence units (RFU) were pooled and concentrated to about 150 μL (100‐kDa NMWCO centrifugal filter) (Merck). The protein concentration was determined by Qubit4 (Thermo Fisher) and samples were stored at −80°C until further use.

### 2.3 Nanoparticle tracking analysis (NTA)

NTA were performed essentially as described by Garcia‐Ceron et al. (2021). Briefly, particle size and concentration were measured using the scatter mode in a ZetaView instrument (Particle Metrix, software 8.05.12 SP1) with a 405 nm laser, which had been calibrated with a solution of 100 nm beads (Thermo Fisher). Samples (1 mL) adjusted to 30–200 particles per frame by dilution in DPBS were injected into the instrument's loading chamber. Eleven chamber positions were measured for data acquisition with a camera sensitivity of 80, shutter speed of 100, minimum and maximum brightness of 30 and 255, respectively, minimum and maximum area of 5 and 1000, respectively, a minimum trace length of 15, and temperature of 25°C. All NTA was performed on EVs that were fresh or stored at −80°C in PBS for up to 2 weeks. All samples were analysed at least in duplicate.

### 2.4 TEM

EV samples were concentrated to 1 μg/μL and 5 μL was prepared as described in (Dawson et al., 2020). Imaging was performed on a Jeol JEM‐2100 electron microscope operating at 200 kV. Images were processed with Gatan Digital Micrograph, version 2.32.888.0.

### 2.5 Glycosidic linkage analysis

Glycosidic linkages were identified and quantified as described by Pettolino et al. (2012), with minor modifications. Briefly, 70 μg of the freeze‐dried EVs (quantified by protein using Qubit) were dissolved in 1 mL of anhydrous dimethyl sulfoxide (DMSO) and methylated by addition of 0.1 mL of methyl iodide under nitrogen followed by sonication for 10 min at room temperature. This step was repeated four times to avoid under‐methylation. One mL of dichloromethane (DCM) was subsequently added to the samples and the methylated polysaccharides were extracted by phase partitioning against deionized water. This step was repeated three times and the resultant combined DCM phases were evaporated under a stream of nitrogen, followed by hydrolysis at 100°C for 3 h in 1 mL of 2 M trifluoroacetic acid (TFA) under nitrogen. The hydrolysates were reduced overnight at room temperature in the presence of NaBD_4_ under nitrogen and acetylated with acetic anhydride at 100°C for 12 h. The partially methylated alditol acetates (PMAAs) were recovered by evaporating the acetic anhydride solvent under a gentle stream of nitrogen and redissolving the samples in DCM. The PMAAs were purified by partitioning against deionized water and the DCM phase was transferred to gas chromatography (GC) vials and analysed on an Agilent 7890B/5977B GC–MS instrument (Agilent Technologies, USA) fitted with a VF‐23 ms capillary column (30 m × 0.25 mm, 0.25 μm, Agilent Technologies, USA). Helium was used as carrier gas and the oven temperature was programmed as follows: from 165 to 175°C at 1°C/min; from 175 to 195°C at 0.5°C/min; from 195 to 210°C at 2°C/min and from 210 to 250°C at 10°C/min, followed by a plateau at 250°C for 6.5 min (total run time 68 min). The fragmentation patterns of the different PMAAs were interpreted by referring to the CCRC Spectral Database for PMAAs (https://glygen.ccrc.uga.edu/ccrc/specdb/ms/pmaa/pframe.html).

### 2.6 Proteomics

Peptides were prepared for mass spectrometry as described by Dawson et al. (2020). EVs (15 μg of protein/biological replicate) were boiled in LDS sample buffer (Life Technologies) and TCEP (tris(2‐carboxyethyl)phosphine) (Thermo Fisher Scientific), and the proteins were separated by short‐range SDS‐PAGE. The gel pieces were excised, and fixed in 50% (v/v) methanol, 7% (v/v) acetic acid for 30 min. The proteins were reduced (2 mM TCEP, 1 h) and alkylated (40 mM iodoacetamide, 30 min in the dark) before proteolysis with 1 μg trypsin (17.8 units/μg) (Promega) for 18 h at 37°C. Peptides were extracted from the gel pieces with 85% (v/v) acetonitrile (ACN), 0.5% (v/v) TFA, lyophilised and resuspended in 20 μL of 5% (v/v) ACN, 0.5% (v/v) TFA. Peptides (1 μg) were injected into an Ultimate 3000 RSLnano UPLC instrument (Thermo Fisher) coupled to a Q‐Exactive HF Orbitrap mass spectrometer (Thermo Fisher). Peptide search was performed using MaxQuant 1.6.3.3, with the label‐free quantitation (LFQ) function and matched against the proteome from the *C. albicans* genome data base using assembly 22 of strain SC5314 (http://www.candidagenome.org/) A 1% false discovery rate (FDR) was applied to the peptide spectrum match and then again at the protein match levels, compared to a decoy data base. The MaxQuant list was processed in Perseus, version v1.6.15.0. Protein identification was only considered positive if more than one peptide was identified across at least two biological replicates.

### 2.7 *G. mellonella* in vivo infection model

The virulence of the fungal strains was assessed using the *in vivo G. mellonella* (wax moth) model (Jemel et al., 2020). The *G. mellonella* larvae were the kind gift of Professor Wieland Meyer (University of Sydney). The larvae were lab reared at 19°C with a 16/8 h light/dark cycle. *C. albicans* isolates were inoculated into 1 mL of YPD and grown overnight with shaking (300 rpm) at 30°C. *C. albicans* cells were pelleted by centrifugation for 2 min at 8000×g and washed three times with 1 mL of sterile DPBS (Gibco). Optical density was measured at 600 nm and cells were diluted in DPBS (Gibco) to a final working OD_600_ of 0.5. *G. mellonella* larvae were starved overnight prior to infection and randomly assigned into each treatment. Larvae were injected with a syringe pump (NE‐1000, Adelab) behind the final proleg with 20 μL of treatment solution using a sterile 29‐gauge needle. EVs used for *in vivo* experiments were stored at −80°C after isolation and thawed on ice prior to inoculation. EVs were mixed with washed *Candida* cells just prior to injection. Treatments were as follows, (1) PBS, (2) PBS with 8.3 ng/μL (protein) of WT EVs, (3) PBS with 8.3 ng/μL (protein) of KO EVs, (4) *C. albicans* WT cells, (5) *C. albicans* WT cells with 8.3 ng/μL (protein) of *sur7Δ* EVs, (6) *C. albicans sur7Δ* cells and (7) *C. albicans sur7Δ*cells with 8.3 ng/μL (protein) of WT EVs. The reciprocal experiment was also performed where *G. mellonella* larvae were infected with *Candida* strains in addition to their own EVs. Treatments were as follows, (1) PBS, (2) PBS with 8.3 ng/μL (protein) of WT EVs, (3) PBS with 8.3 ng/μL (protein) of KO EVs, (4) *C. albicans* WT cells, (5) *C. albicans* WT cells with 8.3 ng/μL (protein) of WT EVs, (6) *C. albicans sur7Δ* cells and (7) *C. albicans sur7Δ* cells with 8.3 ng/μL (protein) of *sur7Δ* EVs.

Larvae were housed in 90 × 25 mm tissue culture plates and incubated at 37°C for 10 days. Survival was recorded daily with dead larvae removed. Statistical analyses were performed in R‐studio using a mixed effects Cox regression by the La Trobe University Statistical Consultancy group.

## 3 RESULTS

**FIGURE 1:**
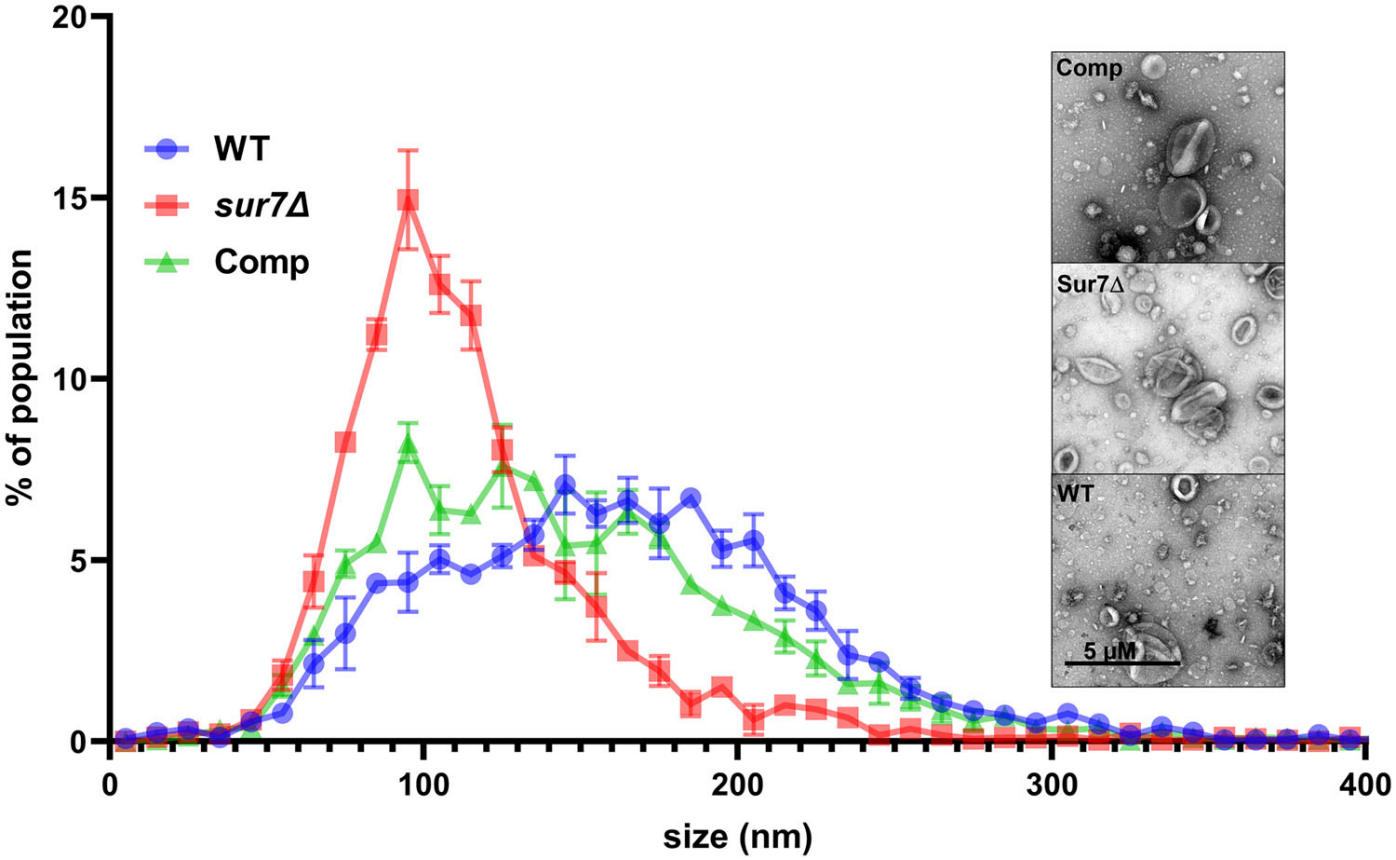
Comparison of EV particle size as determined by nanoparticle tracking analysis. Each line represents the average of the three biological replicates. Error bars are Standard Error of the Mean. Inset‐TEM of EVs, TEM images are not representative of size only EVs morphology.

We have previously reported that Sur7 is significantly enriched in *C. albicans* EVs and is a potential EV marker (Dawson et al., 2020). This led us to consider whether the knockout of *SUR7* would have a detrimental impact on the quantity or characteristics of EVs produced by *C. albicans*. Three EV isolations were thus prepared from each of the three *Candida* strains: DIC185 (WT), YJA11 (*sur7Δ*) and YJA12 (*sur7* complement or comp). The *sur7Δ* strain grew more slowly than either the WT or complement as evidenced by the difference in in final OD, (WT vs. *sur7Δ*, *p* = 0.015) (WT vs. comp, *p* = 0.45) (*sur7Δ* vs. comp, *p* = 0.019) (Table 1). However, there was no difference in total EV protein yield or total number of EVs isolated from the three isolates. There was a significant difference in the mean (WT vs. *sur7Δ*, *p* = 0.0005) (WT vs. comp, *p* = 0.008) (*sur7Δ*v comp, *p* = 0.002) and median EV diameter (WT vs. *sur7Δ*, *p* = 0.0001) (WT vs. comp, *p* = 0.003) (*sur7Δ* vs. comp, *p* = 0.0002) of the three strains. The average diameter of EVs from the *sur7Δ* strain was 30% smaller (116 nm) than EVs from WT (164.2 nm). This phenotype was partially reverted in the complement, where EVs were only 10% smaller (148.2 nm) than WT EVs. (Figure 1). Other than the changes in size, there were no differences in the morphology of the EVs from the different strains under TEM (Figure 1). The difference in size distribution of EVs produced after deletion of *SUR7* suggests that it has a role in biosynthesis and/or morphology of at least one class of EVs produced by *C. albicans*. This led us to investigate whether EVs from these two strains differed in their biochemical composition.

**TABLE 1:** Yield, size and protein content in EVs isolates from WT, *sur7Δ* and Complement lines.

Strain	NTA^a^	1/dilution	Adjusted NTA	Median size (nm)	Mean size (nm)	Starting OD	Final OD	Protein yield (μg)
Comp‐1	9.3 × 10^7^	25000	2.32 × 10^12^	129.9	151.5	0.24	7	53.3
Comp‐2	6.9 × 10^7^	20000	1.38 × 10^12^	135.1	143.9	0.2	7.1	87.6
Comp‐3	3.4 × 10^7^	40000	1.36 × 10^12^	133.7	149.1	0.3	5.15	20.65
		**Average**	**^A^1.68 × 10^12^**	**132.9^A^**	**148.2^A^**	**0.2**	**6.4^A^**	**53.9^A^**
*sur7Δ*−1	5.0 × 10^7^	200000	1.0 × 10^13^	104.6	122.4	0.24	3.2	73.1
*sur7Δ*−2	6.9 × 10^7^	40000	2.76 × 10^12^	102.2	118.3	0.2	3.6	28.4
*sur7Δ*−3	6.4 × 10^7^	40000	2.56 × 10^12^	97.3	109	0.3	4.34	39.4
		**Average**	**^A^5.1 × 10^12^**	**101.4^B^**	**116.6^B^**	**0.2**	**3.7^B^**	**47.0^A^**
WT‐1	7.1 × 10^7^	40000	2.84 × 10^12^	149.4	161.5	0.24	6.9	39.85
WT‐2	4.5 × 10^7^	40000	1.8 × 10^12^	160	168.9	0.2	8.8	73.2
WT‐3	1.8 × 10^8^	20000	3.6 × 10^12^	155.6	162.1	0.3	6.09	33.05
		**Average**	**^A^2.74 × 10^12^**	**155.0^C^**	**164.2^C^**	**0.2**	**7.3^A^**	**48.7^A^**

^a^ Nanoparticle tracking is reported in particles per mL. Superscript letters indicate statistically different groups (*p* < 0.05) within columns.

### 3.1 Deletion of SUR7 decreases virulence in *G. mellonella*, but it can be partially restored by WT EVs

**FIGURE 2:**
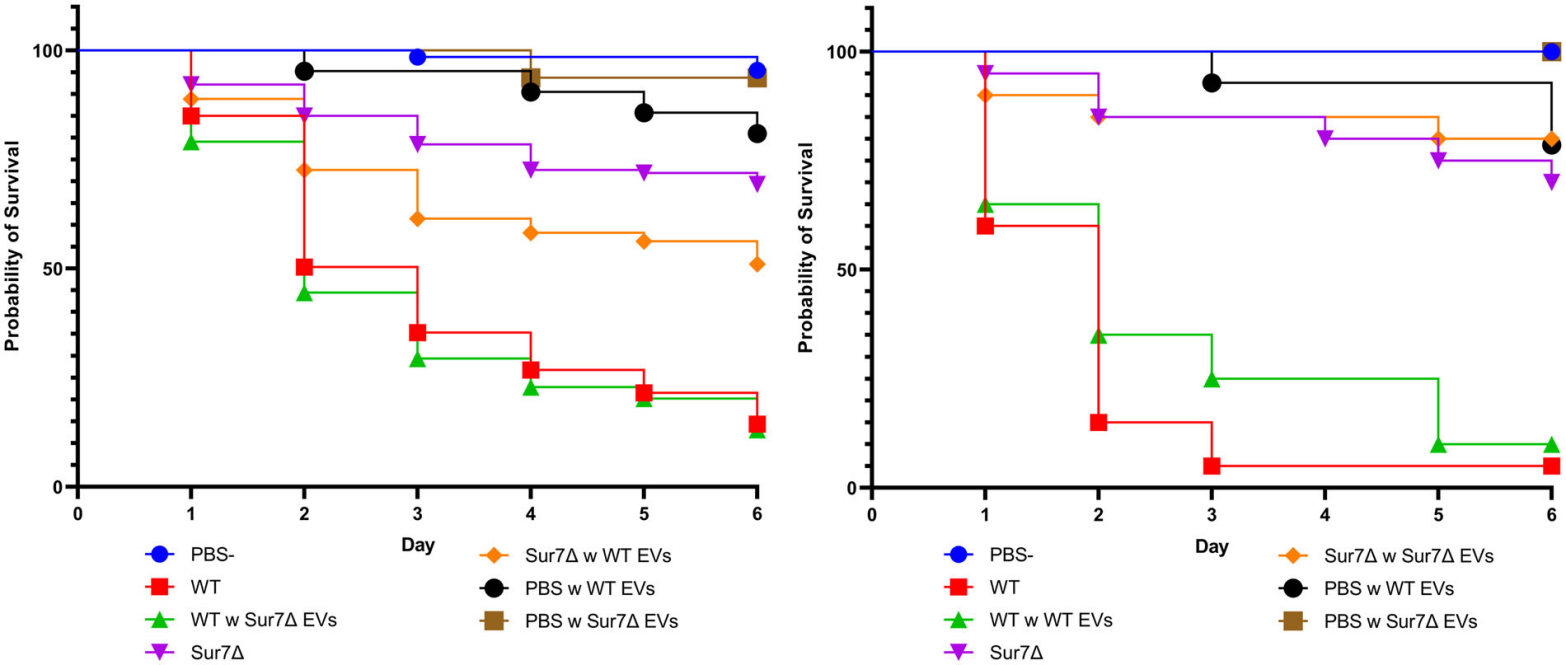
(**Left**) Survival of *G. mellonella* larvae infected with WT and *sur7Δ* lines with or without added WT or *sur7Δ* EVs from opposing lines, 6 days post infection. **N‐** The number of larvae in the pooled experiments. Statistical comparisons (*p* values) between each of the treatments are provided in Table S1. (**Right**) *G. mellonella* larvae infected with WT and *sur7Δ* lines with or without added WT or *sur7Δ* EVs from the same line, 6 days post infection.

The *sur7Δ* strain of *C. albicans* has been reported to be less virulent *in vitro* (macrophage killing assays), and *in vivo* (*G. mellonella* and mice) (Alvarez et al., 2008; Bernardo & Lee, 2010; Douglas & Konopka, 2019; Garcia‐Ceron et al., 2021; Wang et al., 2011, 2016). To assess whether differences in the EVs produced by the *sur7Δ* mutant contribute to the reported virulence defect, six independent *G. mellonella* survival assays were performed. In each assay the larvae received one of seven different treatments; (1) PBS, (2) PBS with WT EVs, (3) PBS with *sur7Δ* EVs, (4) *C. albicans* WT cells, (5) *C. albicans* WT cells with *sur7Δ* EVs, (6) *C. albicans sur7Δ* cells and (7) *C. albicans sur7Δ* cells with WT EVs. The pooled results of these six assays and subsequent *p* values for compared treatments are presented in Figure 2 and Table S1. *G. mellonella* larvae infected with WT *Candida* had a 54% lower survival compared to larvae infected with the *sur7Δ* strain (*p* < 0.0001), confirming the virulence defect reported in the literature. By day six 85% of the larvae infected with WT *Candida* had died compared to 30% of the larvae infected with the *sur7Δ* strain. However, addition of WT EVs to *sur7Δ Candida* partially restored the virulence defect in *sur7Δ* by increasing mortality by approximately 20% over the mortality obtained with the *sur7Δ Candida* alone (*p* = 0.0113). There was no difference in survival at 6 days post infection (*p* = 0.8468) when the *G. mellonella* larvae were infected with either WT *Candida* or WT *Candida* with EVs from the *sur7Δ* line (Figure 2). There was also no difference in survival observed between the PBS or PBS with EV treatments (*p* > 0.3751) (Figure 2). The survival of *G. mellonella* larvae was also compared WT and *sur7Δ* strains with the addition their native EVs (i.e., WT cells with WT EVs and *sur7Δ* cells with *sur7Δ* EVs). Under this scenario no difference was observed in survival between WT cells alone and WT cells with additional EVs or *sur7Δ* cells alone and *sur7Δ* cells with additional EVs (Figure 2).

### 3.2 Proteomic analysis of EVs from Candida strains

**FIGURE 3:**
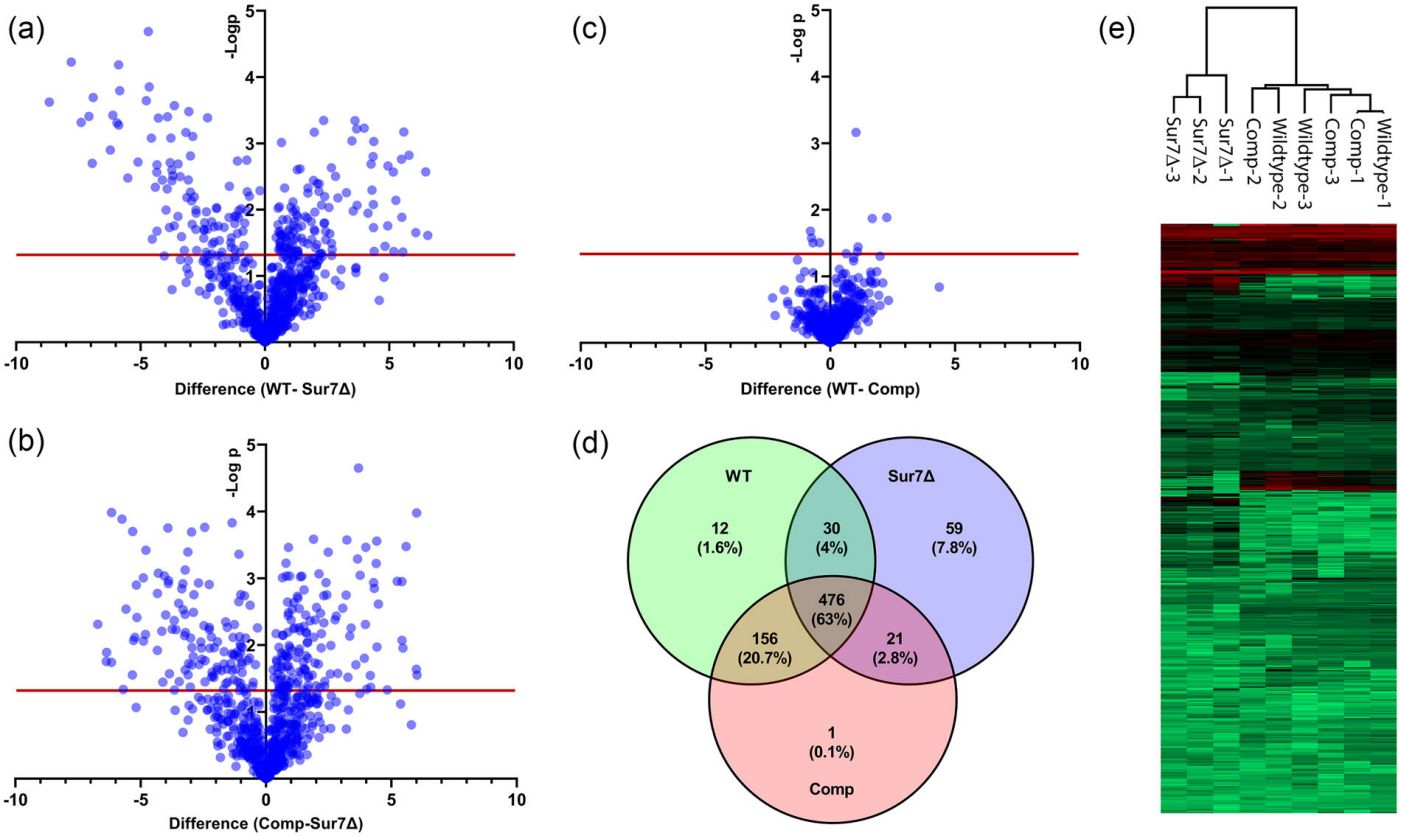
(**a–c)** Volcano plots comparing protein abundance and differential significance between WT, *sur7Δ* and Complement strains, x‐axis is log_2_‐fold change in abundance (red horizontal line indicates *p* = 0.05). (**d**) Venn diagram of proteins observed from each of the *Candida* lines. (**e**) Heat map of the proteomic analysis of the three *Candida* lines.

Given that *C. albicans* WT EVs enhanced the virulence of *sur7Δ* cells in *the G. mellonella* model we compared the protein cargo of the EVs using mass spectrometry to identify proteins that could contribute to virulence. Three biological replicate EV isolations were performed for each of the three *Candida* lines. The proteomes of the EVs from each of the three lines where then compared and the data analysed using Perseus. There were 755 unique proteins identified across the EVs from all strains, with 674, 586 and 654 proteins identified in the EVs from the WT, *sur7Δ* and complement strains, respectively. The heat map generated to visualize the similarity between the biological replicates revealed that the proteomes of the WT and complement lines were more alike (clustering together) than the *sur7Δ* line, which clustered independently (Figure 3e).

Of the 755 proteins identified, 63% (476) were shared by EVs from all three strains. The WT and complement strains shared over 90% of all proteins with each other. In contrast only 67% of proteins were shared between WT and *sur7Δ* EVs. There were 12, 59 and 1 unique proteins identified in the WT, *sur7Δ* and complement strains, respectively (Figure 3d). When only the WT and *sur7Δ* strains were compared, there were 168 and 80 unique proteins, respectively. The 168 proteins unique to WT were examined to determine whether they could contribute to the increased virulence of the WT strain. Volcano plots were used to assess the differences in protein abundance between each of the *Candida* strains (Figure 3a–c). Comparison of the *sur7Δ* EV proteome with the EV proteomes from the complement or WT strains revealed a similar number of proteins that were differentially abundant in each strain (Figure 3a,b). In contrast, there was little difference in protein abundance between the complement and WT strains (Figure 3c).

### 3.3 Functional enrichment of unique proteins from WT and sur7Δ EVs

The unique proteins in the WT and *sur7Δ* EVs were examined further using functional enrichment analysis (FungiFun2) (Priebe et al., 2015). The 80 unique proteins from the *sur7Δ* EVs were associated with three biological processes and two metabolic functions. The overarching function of these unique proteins was transmembrane transport. The biological processes observed were amino acid transmembrane transport (5), siderophore transport (2) and general transmembrane transport (9). The two metabolic processes were lipid binding (4) and protein symporter activity (2). The 80 unique proteins were also ranked in order from most to least abundant. The 10 most abundant unique proteins are listed in Table 2. HSP30 was most the most abundant unique protein and the 19th most abundant protein in *sur7Δ* EVs (Table 2). An extended Table 2 containing the 20 most abundant unique *sur7Δ* EV proteins can be found in the Table S2.

**TABLE 2:** Ten most abundant proteins found in *sur7Δ* EVs but not WT EVs.

Rank	Protein ID	description
19	HSP30	Putative heat shock protein; fluconazole repressed; amphotericin B induced; Spider biofilm induced; rat catheter biofilm induced
24	C6_02330W	Described as a Gag‐related protein; hyphal induced; downregulation correlates with clinical development of fluconazole resistance; repressed by nitric oxide, 17‐beta‐estradiol, ethynyl estradiol
28	CTR1	Copper transporter; transcribed in low copper; induced Mac1, Tye7, macrophage interaction, alkaline pH via Rim101; 17‐beta‐estradiol repressed; complements S. cerevisiae ctr1 ctr3 copper transport mutant; flow model/Spider biofilm induced
45	GAP4	High‐affinity S‐adenosylmethionine permease; required for SAM‐induced morphogenesis; hyphal induced; regulated by Hap43, Gcn2 and Gcn4; colony morphology‐related gene regulation by Ssnp
48	ENA21	Predicted P‐type ATPase sodium pump; Gcn4p‐regulated; flucytosine, amphotericin B, or ketoconazole‐induced; osmotic stress‐induced; overlaps orf19.5170.1, which is annotated as a blocked reading frame; Spider biofilm induced
83	FRE10	Major cell‐surface ferric reductase under low‐iron conditions; seven transmembrane regions and a secretion signal predicted; Tup1, Rim101, Ssn6, Hog1, caspofungin repressed; ciclopirox olamine induced; rat catheter biofilm induced
104	C4_02340W	Putative membrane protein; induced by alpha pheromone in SpiderM medium; Hap4‐induced gene; Spider biofilm induced
128	FRP3	Putative ammonium transporter; upregulated in the presence of human neutrophils; fluconazole‐downregulated; repressed by nitric oxide; Spider biofilm induced; rat catheter biofilm repressed
132	SIT1	Transporter of ferrichrome siderophores, not ferrioxamine B; required for human epithelial cell invasion in vitro, not for mouse systemic infection; regulated by iron, Sfu1, Rfg1, Tup1, Hap43; rat catheter and Spider biofilm induced
133	C1_08900W	Putative lipid raft associated protein; Spider biofilm induced

Proteins are ranked from most abundant to least abundant.

The WT EVs had 168 unique proteins associated with seven statistically enriched biological processes and two metabolic functions. They were β−1,2‐oligomannoside metabolic process (3), proteolysis (12), pathogenesis (17), ubiquitin‐dependent protein catabolic process (11), protein folding (8) cell wall organization (12) and translational termination (4). The metabolic processes were ATP binding (27) and translational termination (4). The 10 most abundant unique proteins are listed in Table 3. ALS4 was the most abundant unique protein, and the 49th most abundant protein in WT EVs (Table 3). An extended Table 3 containing the 20 most abundant unique WT EV proteins can be found in the Table S3.

**TABLE 3:** Ten most abundant proteins found in WT EVs but not *sur7Δ* EVs.

Rank	Protein IDs	Description
49	ALS4	GPI‐anchored adhesin; role in adhesion, germ tube induction; growth, temperature regulated; expressed during infection of human buccal epithelial cells; repressed by vaginal contact; biofilm induced; repressed during chlamydospore formation
58	RBE1	Pry family cell wall protein; Rim101, Efg1, Ssn6, alkaline repressed; O‐glycosylation; no GPI anchor predicted; ketoconazol induced; regulated by Sef1, Sfu1, Hap4; flow model biofilm induced; rat catheter and Spider biofilm repressed
78	PLB4.5	Phospholipase B; Hog1‐induced; regulated by Ssn6; putative GPI‐anchor; repressed during cell wall regeneration; clade‐associated gene expression; Hap43‐induced; rat catheter and Spider biofilm repressed
89	C2_10150W	Secreted protein; fluconazole‐induced
91	KEX2	Subtilisin‐like protease (proprotein convertase); processes aspartyl proteinase Sap2; required for hyphal growth and wild‐type virulence in mice; required for maturation of candidalysin Ece1p
118	CHT1	Chitinase; putative N‐terminal catalytic domain; has secretory signal sequence; lacks S/T region and N‐glycosylation motifs of Chs2p and Chs3p; alkaline downregulated; expression not detected in yeast‐form or hyphal cells
134	RBT1	Cell wall protein with similarity to Hwp1; required for virulence; predicted glycosylation; fluconazole, Tup1 repressed; farnesol, alpha factor, serum, hyphal and alkaline induced; Rfg1, Rim101‐regulated
137	ADH2	Alcohol dehydrogenase; soluble in hyphae; expression regulated by white‐opaque switching; regulated by Ssn6; indued by Mnl1 in weak acid stress; protein enriched in stationary phase yeast cultures; Spider biofilm induced
148	GDA1	Golgi membrane GDPase, required for wild‐type O‐mannosylation, not N‐glycosylation; required for wild‐type hyphal induction, cell wall, and cell surface charge; not required for HeLa cell adherence; functional homolog of S. cerevisiae Gda1p
225	RPN2	Putative 26S proteasome subunit; transcript regulated by Mig1; caspofungin repressed; regulated by Gcn2 and Gcn4; gene used for strain identification by multilocus sequence typing

Proteins are ranked from most abundant to least.

**TABLE 4:** Twenty most differentially abundant proteins *sur7Δ* EVs compared to WT.

Fold difference	Name	Description
408	CDR1	Multidrug transporter of ABC superfamily; transports phospholipids in an in‐to‐out direction; induced by beta‐estradiol, progesterone, corticosteroid, or cholesterol; Spider biofilm induced
221	PHM7	Putative transporter, possibly involved in ion homeostasis, drug tolerance, filamentous growth, virulence; fungal‐specific; Hog1‐repressed; repressed by 17‐beta‐estradiol, ethynyl estradiol; Hap43‐induced; Spider biofilm induced
168	HSP30	Putative heat shock protein; fluconazole repressed; amphotericin B induced; Spider biofilm induced; rat catheter biofilm induced
135	CTR1	Copper transporter; transcribed in low copper; induced Mac1, Tye7, macrophage interaction, alkaline pH via Rim101; 17‐beta‐estradiol repressed; complements S. cerevisiae ctr1 ctr3 copper transport mutant; flow model/Spider biofilm induced
123	FTR1	High‐affinity iron permease; required for mouse virulence, low‐iron growth; iron, amphotericin B, caspofungin, ciclopirox, Hog1p, Sef1p, Sfu1p, and Hap43p regulated; complements S. cerevisiae ftr1 iron transport; Hap43p‐repressed
120	C6_02330W	Described as a Gag‐related protein; hyphal induced; downregulation correlates with clinical development of fluconazole resistance; repressed by nitric oxide, 17‐beta‐estradiol, ethynyl estradiol
62	FRE10	Major cell‐surface ferric reductase under low‐iron conditions; seven transmembrane regions and a secretion signal predicted; Tup1, Rim101, Ssn6, Hog1, caspofungin repressed; ciclopirox olamine induced; rat catheter biofilm induced
59	SSO2	Plasma membrane t‐SNARE; involved in fusion of secretory vesicles at the plasma membrane
69	GAP4	High‐affinity S‐adenosylmethionine permease; required for SAM‐induced morphogenesis; hyphal induced; regulated by Hap43, Gcn2 and Gcn4; colony morphology‐related gene regulation by Ssnp
75	HGT6	Putative high‐affinity MFS glucose transporter; 20 family members; induced in core stress response; fluconazole, oropharyngeal candidasis induced; flow model biofilm induced; Spider biofilm induced
57	CFL2	Oxidoreductase; iron utilization; Sfu1/Sef1/Hap43/Nrg1/Tup1/Rim101 regulated; alkaline/low iron/fluphenazine/ciclopirox olamine, flucytosine, fluconazole, Spider/flow model/rat catheter biofilm induced; caspofungin/amphotericin B repressed
59	ENA21	Predicted P‐type ATPase sodium pump; Gcn4p‐regulated; flucytosine, amphotericin B, or ketoconazole‐induced; osmotic stress‐induced; overlaps orf19.5170.1, which is annotated as a blocked reading frame; Spider biofilm induced
46	HGT1	High‐affinity MFS glucose transporter; induced by progesterone, chloramphenicol, benomyl; likely essential for growth; protein newly produced during adaptation to the serum; rat catheter and Spider biofilm induced
34	CIP1	Possible oxidoreductase; transcript induced by cadmium but not other heavy metals, heat shock, yeast‐hypha switch, oxidative stress (via Cap1), or macrophage interaction; stationary phase enriched protein; Spider biofilm induced
27	RSN1	Putative membrane protein; induced by alpha pheromone in SpiderM medium; Hap4‐induced gene; Spider biofilm induced
25	ZRT1	Putative zinc transporter; acts with Pra1 in sequestration of zinc from host tissues during infection; hyphal, macrophage‐induced; alkaline induced upon adherence to polystyrene; induced in oropharyngeal candidasis; Spider biofilm induced
26	FRP3	Putative ammonium transporter; upregulated in the presence of human neutrophils; fluconazole‐downregulated; repressed by nitric oxide; Spider biofilm induced; rat catheter biofilm repressed
24	C1_00310W	Putative protein of unknown function; shows colony morphology‐related gene regulation by Ssn6p
21	NCE102	Non classical protein export protein; localized to plasma membrane; Hap43‐induced gene; flow model biofilm induced; Spider biofilm induced
20	GSC1	Essential beta‐1,3‐glucan synthase subunit; gsc1 allele determines resistance/sensitivity to echinocandins; 16 predicted membrane‐spanning regions; mRNA abundance declines after yeast‐to‐hypha transition; Spider biofilm induced

Shaded proteins were not observed in WT EVs.

### 3.4 Analysis of relative protein abundance

To assess which proteins may have the most functional importance, relative levels of protein abundance between *sur7Δ* EVs and WT EVs were compared. There were 47 proteins that were statistically more than 2‐fold more abundant in WT EVs than *sur7Δ* EVs, and the *sur7Δ* EVs had 57 proteins that were at least 2‐fold more abundant than in the WT EV proteome. The 20 proteins that were most enriched in WT versus *sur7Δ* EVs are presented in Table 4 and the 20 proteins that were most enriched in *sur7Δ* versus WT EVs are listed in Table 5.

**TABLE 5:** Twenty most differentially abundant proteins in WT EVs compared to *sur7Δ*.

Fold change	Protein IDs	Description
88	ENG1	Endo‐1,3‐beta‐glucanase; ortholog of *S. cerevisiae* Dse4 needed for cell separation; caspofungin, fluconazole repressed; repressed by alpha pheromone in SpiderM medium; flow model biofilm induced; rat catheter biofilm repressed
59	RBT4*	Pry family protein; required for virulence in mouse systemic/rabbit corneal infections; not filamentation; mRNA binds She3, is localized to hyphal tips; Hap43‐induced; in both yeast and hyphal culture supernatants; Spider biofilm induced
55	ALS4	GPI‐anchored adhesin; role in adhesion, germ tube induction; growth, temperature regulated; expressed during infection of human buccal epithelial cells; repressed by vaginal contact; biofilm induced; repressed during chlamydospore formation
45	MP65*	Cell surface mannoprotein; cell‐wall glucan metabolism, adhesion; adhesin motif; O‐glycosylation; induced by heat, germ tube formation, wall regeneration; mycelial antigen; diagnostic marker; fluconazole‐repressed; Spider biofilm induced
45	PLB4.5	Phospholipase B; Hog1‐induced; regulated by Ssn6; putative GPI‐anchor; repressed during cell wall regeneration; clade‐associated gene expression; Hap43‐induced; rat catheter and Spider biofilm repressed
38	RBE1*	Pry family cell wall protein; Rim101, Efg1, Ssn6, alkaline repressed; O‐glycosylation; no GPI anchor predicted; ketoconazol induced; regulated by Sef1, Sfu1, Hap4; flow model biofilm induced; rat catheter and Spider biofilm repressed
36	XOG1	Exo‐1,3‐beta‐glucanase; five glycosyl hydrolase family member; affects sensitivity to chitin and glucan synthesis inhibitors; not required for yeast‐to‐hypha transition or for virulence in mice; Hap43‐induced; Spider biofilm induced
33	BGL2*	Cell wall 1,3‐beta‐glucosyltransferase; mutant has cell‐wall and growth defects, but wild‐type 1,3‐ or 1,6‐beta‐glucan content; antigenic; virulence role in mouse systemic infection; rat catheter biofilm induced
31	IFO3	Secreted protein; fluconazole‐induced
21	RBT1*	Cell wall protein with similarity to Hwp1; required for virulence; predicted glycosylation; fluconazole, Tup1 repressed; farnesol, alpha factor, serum, hyphal and alkaline induced; Rfg1, Rim101‐regulated
20	KEX2*	Subtilisin‐like protease (proprotein convertase); processes aspartyl proteinase Sap2; required for hyphal growth and wild‐type virulence in mice; required for maturation of candidalysin Ece1p
20	CHT1	Chitinase; putative N‐terminal catalytic domain; has secretory signal sequence; lacks S/T region and N‐glycosylation motifs of Chs2p and Chs3p; alkaline downregulated; expression not detected in yeast‐form or hyphal cells
19	ADH2	Alcohol dehydrogenase; soluble in hyphae; expression regulated by white‐opaque switching; regulated by Ssn6; indued by Mnl1 in weak acid stress; protein enriched in stationary phase yeast cultures; Spider biofilm induced
18	YOS9	Ortholog(s) have oligosaccharide binding activity and role in endoplasmic reticulum unfolded protein response, retrograde protein transport, ER to cytosol, ubiquitin‐dependent ERAD pathway, ubiquitin‐dependent glycoprotein ERAD pathway
16	GDA1	Golgi membrane GDPase, required for wild‐type O‐mannosylation, not N‐glycosylation; required for wild‐type hyphal induction, cell wall, and cell surface charge; not required for HeLa cell adherence; functional homolog of S. cerevisiae Gda1p
13	IFO2	Secreted protein; fluconazole‐induced
13	LHS1	Protein similar to S. cerevisiae Hsp70p; predicted Kex2p substrate; possibly essential, disruptants not obtained by UAU1 method; flow model biofilm repressed
12	RPN2	Putative 26S proteasome subunit; transcript regulated by Mig1; caspofungin repressed; regulated by Gcn2 and Gcn4; gene used for strain identification by multilocus sequence typing
10	CYP5	Putative peptidyl‐prolyl cis‐trans isomerase; macrophage‐downregulated protein level; protein level decrease in stationary phase cultures; predicted endoplasmic reticulum (ER) localization
8	URA2	Putative bifunctional carbamoylphosphate synthetase‐aspartate transcarbamylase; flucytosine induced; macrophage/pseudohyphal‐induced; 5′‐UTR intron; flow model biofilm repressed

* Pathogenesis associated proteins, Shaded proteins were not observed in *sur7Δ* EVs.

### 3.5 Functional enrichment of differentially abundant proteins

FungiFun was used to determine if there was any functional enrichment in the 57 proteins that were more than two‐fold more abundant in *sur7Δ* EVs compared to WT EVs. The most statistically significant metabolic functions were metal ion binding, glucanosyltransferase activity, xenobiotic‐transporting ATPase activity and calcium‐transporting ATPase activity. The most statistically significant biological functions were high‐affinity iron ion transmembrane transport and cellular response to oxidative stress (*p* < 0.01). Of the 47 proteins that were more than two‐fold more abundant in WT EVs compared to *sur7Δ* EVs the most statistically significant metabolic processes were glucan exo‐1,3‐beta‐glucosidase and glucan endo‐1,3‐beta‐D‐glucosidase activity, the most statistically significant biological functions were cellular glucan metabolic process and establishment or maintenance of cell polarity regulating cell shape (*p* > 0.01).

If we focus on only the top 20 most differentially abundant *sur7Δ* EVs proteins (Table 4) the five most notable biological processes are iron ion transport, protein secretion, glucose transport, carbohydrate transport and azole transport (*p* > 0.05). In WT EVs the 20 most differentially abundant proteins are associated with cellular glucan metabolic process, pathogenesis, cell‐substrate adhesion, single‐species biofilm formation in or on host organism and fungal‐type cell wall organization (*p* > 0.001). There was a significant overlap in the proteins in each of those biological process categories, for example XOG1 is present in all but pathogenesis, and pathogenesis‐related proteins are present at least once in all of the five biological processes listed above. Indeed, six of the top 11 most abundant WT proteins are pathogenesis related (Table 5).

### 3.6 Effect of sur7Δ on the carbohydrate composition of *C. albicans* EVs

**FIGURE 4:**
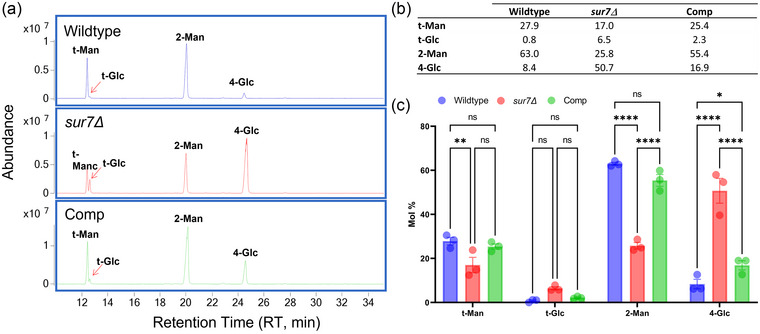
Glycosidic linkage analysis of carbohydrates in EVs from WT (DIC185), the *sur7Δ* (YJA11) and the *sur7Δ* complement (YJA12) strains. (**a**) Representative linkage analysis gas chromatogram. (**b**) Relative levels of observed carbohydrate linkages (**c**) Relative amounts of carbohydrate linkages with statistical analysis. t‐(terminal), Man (mannose), Glc (glucose), 2‐Man (1,2‐mannosyl residues) and 4‐Glc (1,4‐glucosyl residues) are presented. Linkage types were confirmed by Electron‐impact MS on the permethylated alditol acetates (fragmentation data is supplied supplementary material).

Given that the proteomic analysis identified a number of differentially abundant proteins known to be either heavily glycosylated (MP65) or involved in carbohydrate synthesis (ENG1) we analysed the carbohydrate composition of EVs from each strain. Glycosidic linkage analysis revealed substantial differences in the carbohydrate composition of EVs from the WT, the *sur7Δ* and the *sur7Δ* complement strains (Figure 4). EVs from the *sur7Δ* strain contained approximately half the 1,2‐mannosyl residues (2‐Man) and three‐fold more 1,4‐Glucosyl residues (4‐Glc) than EVs from the WT strain. The complemented strain had 2‐Man and 1,4‐Glc levels that were intermediate to the WT and *sur7Δ*. The *sur7Δ* and complement lines both had small amounts of terminal glucosyl residues (t‐Glc), that was not observed in the WT. The *sur7Δ* strain had a reduction in terminal mannosyl residues (t‐Man) compared to the WT.

## 4 DISCUSSION

In an earlier study (Dawson et al., 2020), we discovered that the plasma membrane protein *sur7Δ* is abundant and enriched in EVs produced by *C. albicans* and is a potential EV marker. Sur7 is a component of the MCC/eisosome complex which is a site of endocytosis. This led to the question of whether this complex or Sur7 has a role in EV production. We compared EVs from WT, the *sur7Δ* strain and a *SUR7* complement of the deletion strain to determine whether *SUR7* deletion affected EV production, morphology, protein or carbohydrate cargo and finally whether the EVs had any impact on the virulence of *C. albicans*.

### 4.1 EVs from sur7Δ are smaller than WT EVs

EVs from the *SUR7* KO were smaller than EVs from WT. The EVs isolated from the WT strain based on SC5314 had a mean diameter of 164 nm. Comparatively EVs from the complement and *sur7Δ* were 10% and 30% smaller, respectively. Thus, deletion of *sur7* led to a physical change to the EV size distribution. The size of the EVs from our WT line is similar to the size of EVs from other *C. albicans* strains as well as EVs isolated from biofilms where the size ranged from 50 to 250 nm (Dawson et al., 2020; Zamith‐Miranda et al., 2021). The number of EVs and the total proteins isolated from the WT EVs (based on SC5314) *Candida* strain were similar to those published for other *C. albicans* strains (Dawson et al., 2020; Piffer et al., 2021).

There are several potential explanations for the decrease in size of EVs from the *sur7Δ* line. Firstly, *Candida* is likely to produce different classes of EVs, as occurs in mammalian cells (Dechantsreiter et al., 2022; Shen et al., 2018). The classes may be produced by different biosynthetic pathways and carry distinctive cargo that relates to the range of biological functions. Fractionation techniques such as those used for mammalian EVs should be employed to establish whether *C. albicans* produces different classes of EVs and to determine whether *sur7* deletion blocks or enhances production of specific classes. EV populations are known to be heterogenous, perhaps Sur7 is involved in biogenesis/release of a specific subset of EVs that are related to virulence and/or the larger end of the EV size spectrum (Rizzo et al., 2020). Alternatively, Sur7 maybe involved in the curation of the cargo of larger EVs, without Sur7, larger EVs are no longer produced. Another explanation is that knockout of Sur7 affects the membrane curvature and changes the properties of the membrane, reducing the size of the EVs produced. Sur7 has a similar four transmembrane domain topology to the tetraspanins from mammalian cells, which have been implicated in modulating membrane curvature (Umeda et al., 2020). Thus, Sur7 might function in a similar role in *Candida* EVs, the knockout of which reduces the ability of the fungi to produce EVs of a larger diameter. Knockout of the gene encoding the CD9 tetraspanin in bone marrow dendritic cells (BMDCs) has been reported to decrease release of exosomes to one third the levels of WT, but the size distribution of the exosome population was not compared (Chairoungdua et al., 2010). Likewise, knockout of the tetraspanin CD63 in HEK293 cells, decreased EV production and resulted in fewer small vesicles (Hurwitz et al., 2016). In our study we did not observe a reduction in the number of EVs after *SUR7* deletion.

### 4.2 Role of *C. albicans* EVs in infection

In our study the addition of EVs during the fungal infection process enhanced lethality of the *sur7Δ Candida* line in *G. mellonella in vivo*. In contrast, addition of WT EVs to WT *Candida* cells did not enhance virulence. This suggests that WT EVs carry virulence factors that are important for the infection process and are reduced or missing in *sur7Δ* EVs.

EVs are taken up by macrophages and initiate the innate defence response via toll receptors (Vargas et al., 2015). EVs are not lethal in their own right, but addition of EVs at the same time as the pathogen has been reported to enhance fungal burden during infection by *C. neoformans*. Indeed, Huang et al. (2012) demonstrated that 2–3 times more *C. neoformans* cells entered the brain or cerebrospinal fluid of mice within 24 h when the infection was performed concomitantly with 50 μg or 100 μg of EVs (by protein content) (Huang et al., 2012). Similarly, Marina and co‐workers observed a higher burden of *C. neoformans* in the lungs of mice 15 DPI, when EVs were administered intranasally after infection on days two and four (Marina et al., 2020). Conversely pre‐challenge or “immunisation” with EVs prior to challenge with the pathogen can protect against infection. EVs from *Cryptococcus* and *Candida* improved survival of *Galleria* larvae when the larvae were challenged with EVs alone 2 days prior to infection (Colombo et al., 2019; Vargas et al., 2015). Furthermore, this protection was replicated in a murine model, where fungal burden and mortality was reduced when EVs were administered four times 1 week apart, prior to infection (Vargas et al., 2020). In each of these models the EVs had stimulated the immune system prior to infection, thus establishing the immunogenicity of EVs. In contrast, Ikeda and co‐workers used a murine subcutaneous model with the fungal pathogen *Sporothrix brasiliensis*, in which they injected EVs twice at 7‐day intervals before infection with the pathogen (Ikeda et al., 2018). The pre‐inoculation resulted in higher fungal burden and increased lesion size (Ikeda et al., 2018). Octaviano and co‐workers obtained similar results after inoculating three times, with EVs at 7‐day intervals prior to infection (Octaviano et al., 2022). The results from this model and pathogen do not reflect the work of others using *C. albicans* and *C. neoformans*, where a “vaccination” effect was observed (Colombo et al., 2019; Vargas et al., 2015; Vargas et al., 2020). These contradicting results are likely to reflect the heterogeneity of the EV populations used, the amount used, the production source and EV age/storage conditions. For example, in some experiments EVs that had been stored at 4°C produced an immune response but were less effective than freshly isolated EVs (Vargas et al., 2020).

In our study the addition of EVs during the fungal infection process directly contributed enhanced lethality *in vivo*. Hence, we set out to identify the components of EV cargo that could contribute to the virulence of *C. albicans* EVs in fungal infection.

### 4.3 sur7Δ EVs lack important pathogenesis‐related proteins compared to WT EVs

Proteomic analysis of EVs from WT, *sur7Δ* and complement identified a total of 755 unique proteins. Comparison of the EVs from the three strains revealed substantial differences in the protein cargos. The WT EVs had a protein population consistent with that we have reported previously (Dawson et al., 2020). The proteomes of the EVs from WT and complement strains were similar, in contrast to the proteome of the EVs from the *sur7Δ* strains. Six of the top 11 most differentially abundant (WT vs. *sur7Δ*) EV proteins from the WT strain were associated with pathogenesis. They are the pry family proteins RBT4 and RBE1, the cell wall or cell surface associated proteins MP65, BGL2 and RBT2, and KEX2, a Subtilisin‐like protease required for the maturation of a candidalysin. RBE1 and RBT4 are secreted by *C. albicans* cells (Röhm et al., 2013) and have predicted signal sequences. Knockout of either gene reduced virulence in mouse systemic infection models, and the double knockout impacted virulence synergistically (Röhm et al., 2013). The double knockout strain was also more susceptible to human neutrophil cells, similar to the observation of increased susceptibility of the *sur7Δ* strain in macrophage killing assays (Bernardo & Lee, 2010). KEX2 has an important role in the virulence of *Candida*. The KEX2Δ *Candida* line has reduced ability to kill macrophages *in vitro* and has attenuated virulence in the mouse model of systemic *Candida* infection (Newport et al., 2003). Zarnowski and co‐workers also detected KEX2 in EVs produced by biofilms but not by planktonic cells (Zarnowski et al., 2018). Dawson et al. also identified KEX2 in EVs from *Candida* biofilms as well as planktonic cells. In both cases it was detected exclusively in the EVs and not in WCL (Dawson et al., 2020). Interestingly, other predicted substrates of KEX2 are among the 20 most abundant proteins in WT EVs, including XOG1 and LHS1, and the pathogenesis‐related proteins MP65 and RBT1 (Newport et al., 2003). Deletion of the MP65 gene causes severe defects in biofilm formation (Sandini et al., 2011). Likewise, both BGL1 and XOG1 are important for biofilm formation and are present in EVs produced by *Candida* cells growing in either planktonic or biofilm forms (Taff et al., 2012; Zarnowski et al., 2018). Thus the 45, 36 and 33‐fold reductions of these proteins in *sur7Δ* EVs may explain at least in part why the *sur7Δ* line is poor at forming biofilms (Bernardo & Lee, 2010).

No known pathogenesis‐related proteins were present in the 10 most abundant unique or the differentially abundant proteins in the *sur7Δ* EVs. Notably, the pathogenesis‐related proteins, RBE1 and KEX2 were not detected in *sur7Δ* EVs, nor were the proteins that are present in WT EVs that are activated by KEX2 (RBT1 and LHS1). Other pathogenesis‐related proteins were either absent or greatly reduced in abundance in *sur7Δ* EVs relative to WT (RBT4, MP65, XOG1). This may explain the greater susceptibility to macrophages and the reduction in virulence of the *sur7Δ* line. It would be interesting to assess whether WT EVs restore virulence of the RBT4, RBE1 and KEX2 knockout lines (the LHS1 knockout is not available). Of the potential virulence factors listed in Table 5, only RBT4 has been reported to change expression in WCL (5X up or down), where expression increased by 11‐fold in *sur7Δ* WCL compared to wild type (Alvarez et al., 2008). We detected RBT4 in only one of the replicates of the *sur7Δ* EVs and this was at very low levels.

### 4.4 The deletion of SUR7 leads to the incorporation of membrane transporters

The top 20 most differentially abundant proteins in the EVs of *sur7Δ* compared to WT included several membrane proteins involved in drug, ion and glucose transport. Sur7 has an important role in plasma membrane organisation (Wang et al., 2016) explaining why deletion of *SUR7* may result in loss of transporters from the membrane and incorporation into EVs. An important example of aberrant location of a membrane protein is NCE102 which was present in EVs from the *sur7Δ* strain but not in WT EVs or the complemented strain. NCE102 is also a member of the MCC complex, like Sur7. Thus, Sur7 appears to be important for regulation of EV protein cargo in fungi, as the tetraspanins are for regulation of cargo in mammalian systems (Jankovičová et al., 2020).

Several genes have been reported to be expressed five times more highly in the *sur7Δ* than the WT strain (Alvarez et al., 2008). However, in this study the encoded protein of only two genes, (*RBT4* and *ECM331*‐ A GPI anchored protein of unknown function), were more abundant in *sur7Δ* EVs versus WT EVs. The expression of ECM331 was 26‐fold higher in *sur7Δ* cells (Alvarez et al., 2008). ECM331 was observed in the EVs of *sur7Δ* and complement strains but not in WT. Thus, gene products that are overexpressed in the cells of the *sur7Δ* line (relative to WT) are not necessarily abundant in *sur7Δ* EVs.

### 4.5 Mispackaging of the CDR1 in sur7Δ EVs may enhance susceptibility to azoles

CDR1 was 400‐fold more abundant in the *sur7Δ* EVs, compared to WT EVs. CDR1 was identified in WT EVs from all *C. albicans* strains assessed by Dawson and co‐workers including those grown as a biofilm, and was also enriched in EVs compared to WCL (Dawson et al., 2020). The overexpression of CDR1 is associated with azole resistance in *Candida* (Siikala et al., 2010), so the mispackaging of excess CDR1 to EVs could lead to a depletion of cellular CDR1 and a reduction in azole resistance. Thus, the overabundance of the CDR1 in the *sur7Δ* EVs may explain the observation that the *sur7Δ* strain is more susceptible to fluconazole than the WT (Douglas et al., 2012).

### 4.6 The protein cargo of Fungal EVs is conserved across fungal isolates and growth conditions

Just over 40% of the proteins identified in WT EVs of our strain SC5314 based) were also detected in the EVs of DAY286 (a closely related WT strain) from an unrelated experiment (Dawson et al., 2020). This number compares well with the report that about 50% of observed proteins were shared in the EVs of DAY286 and ATCC10231 when compared directly by Dawson et al. under the same growth conditions (Dawson et al., 2020).

The observed differences in protein cargo between this and previous studies can be attributed to the use of different isolates, culture in different medium (YPD vs. RPMI) and a different isolation method (size exclusion vs. ultracentrifugation). From this comparison we can conclude that size exclusion is an effective method of isolating EVs from *C. albicans*. In our study there was 90% similarity between the WT and complement EV proteomes. Similarity was reduced to 67% when the *SUR7* gene was deleted. Therefore, *Candida* mutants which alter the cargo of EVs, or their EV production machinery, offer a lucrative opportunity to better interrogate the function of these particles and how they contribute to the biology of fungi.

### 4.7 EVs from sur7Δ have reduced 1,2‐mannosyl residues and elevated levels of a glycogen‐like molecule

EVs from *sur7Δ* have a different carbohydrate composition to WT EVs. The *sur7Δ* EVs had slightly fewer terminal mannosyl residues, fewer 1,2‐linked mannosyl residues (<50%) and a 6‐fold increase in 1,4‐glucosyl residues. There are few reports on the carbohydrate cargo of fungal EVs and its potential role.

The carbohydrate analysis used here does not provide any information on anomery, that is, α or β linkages, as anomery is lost during the chemical modification used for linkage analysis. Hence the 1,4‐glucosyl linkages detected could arise from a cytosolic glycogen‐like α−1,4‐glucose polymer or a cell wall pseudo‐cellulose β−1,4‐linked polymer. There are very few reports in the literature of cellulose‐like β−1,4‐glucans in fungi, with one example being the linear β−1,4‐glucan reported to be secreted by the snow mould fungus, *Microdochium nivale* (Schweiger‐Hufnagel et al., 2000). However, there are a few examples of fungal α−1,4‐glucans described in the literature that are similar to glycogen. *C. albicans* builds cytosolic glycogen stores towards the end of exponential growth and consumes them when carbohydrates and nutrients become limiting (Zeitz et al., 2019). However, glycogen‐like polysaccharides typically contain α−1,6‐glucosidic linkages, forming branched structures, in addition to an α−1,4‐linked glucosyl residues. In our analyses, no α−1,6‐linked glucosyl residues were detected, indicating that, if present these would be below detectable levels with the method used here (Zeitz et al., 2019). Furthermore, glycogen typically forms 20 nm cytosolic particles which would consume significant volume within an EV. Potential glycogen‐like fragments composed of α−1,4‐glucans and single substitutions of α−1,6‐Glc*p* residues along the main chain have been identified in *Paracoccidioides* EVs (Peres da Silva, Heiss et al., 2015). The authors of this report proposed that glycogen and/or its hydrolysis products are transported within EVs to the cell wall and extracellular environment. In another example, Bittencourt and co‐workers reported a glycogen‐like polysaccharide consisting of linear α−1‐4‐Glc*p* residues with α−1‐6‐Glc substitutions from *Pseudallescheria boydii* that stimulates cytokine secretion by cells of the innate immune system via the toll‐like receptor 2 and blocks phagocytosis in a dose‐dependent manner (Bittencourt et al., 2006). Likewise *sur7Δ Candida* cells which have elevated levels of 1,4‐linked glucosyl residues in their EVs compared to WT are less efficiently phagocytosed than WT cells (Douglas et al., 2012). Some α−1,4‐Glc*p* residues have been observed in the cell walls of several other fungal species; however, in our analysis we did not observe any evidence of other carbohydrate linkages that are usually associated with the cell wall (i.e., sugar derivatives arising from chitin and β−1,3/6‐glucans) (Ruiz‐Herrera et al., 2006).

The mannosyl residues present in *C. albicans* glycoproteins typically arise from polysaccharides that consist of an α−1,6‐linked mannan main chain with predominantly α−1,2‐ and some α−1,3‐linked side chains. They can also contain α−1,6‐branched and β−1,2‐linked mannosyl residues (Shibata et al., 2012). The high mannose content observed in our samples suggests the WT EVs carry glycoproteins. This is consistent with the results of Rizzo and co‐workers who used cryo‐EM to demonstrate that ∼90% of *C. neoformans* EVs are covered by a mannoprotein‐based fibrillar material. They observed similar adornment on the surface of *C. albicans* EVs and hypothesized that the enrichment of tetraspanin‐like membrane proteins containing a Sur7/PalI family motif in EVs indicates that decorated EVs could be specifically shed from Sur7 specialized plasma membrane domains (Rizzo et al., 2021). Interestingly they also observed that the decorated EVs are normally larger in diameter than undecorated EVs. This observation mirrors our results, where EVs from the *sur7Δ* line with the lower levels of 1,2‐mannosyl residues were smaller than EVs from WT. Thus, a cryo‐EM approach as taken by Rizzo and co‐workers for the analysis of *sur7Δ* EVs would be an effective way to reveal the presence or absence of this carbohydrate adornment (Rizzo et al., 2021).

The reduction in 1,2‐mannosyl residues in the *sur7Δ* EVs may be explained by the absence of three proteins from the *sur7Δ* line EVs which are involved in β−1,2‐oligomannoside metabolism (BMT3 BMT4 & RDH1). Although these proteins were not observed in the *sur7Δ* EVs, their abundance in WT was very low and thus they were not considered as differentially abundant, and there is currently no evidence that oligosaccharide synthesis occurs in EVs.

The reduction in the observed amount of mannosyl residues in the *sur7Δ* EVs is an interesting counterpoint as the cell walls of *sur7Δ* cells have increased levels of these types of residues (Wang et al., 2011). One important mannoprotein is MP65, which in *Candida* is an abundant cell surface proteoglycan with predicted O‐glycosidic linkage sites. The purified form of this protein induces T‐cell proliferation of human peripheral blood mononuclear cells *in vitro* (Gomez et al., 1996). In WT EVs, it is 45‐fold more abundant than in *sur7Δ* EVs and thus may explain in part the large reduction in mannosyl residues observed in *sur7Δ* EVs. Furthermore MP65 stimulates the release of cytokines via the activation of Toll‐like receptors 2 and 4 (TLR2 & TLR4) (Pietrella et al., 2006). MP65 has been investigated as both a diagnostic tool for *Candida* infections and a potential vaccination strategy (Zito et al., 2016). Likewise, Bgl2 (1,3‐β‐glucosyltransferase) was also observed to be more than 30‐fold more abundant in WT EVs than in *sur7Δ* EVs. This protein has been investigated as an antigen and vaccine candidate, and the treatment of mice with Bgl2 in a vaccine model increased survival of mice challenged with *C. albicans* (Gil‐Bona et al., 2015). Thus, we speculate that the reduction in Bgl2 in EVs of the *sur7Δ* line means that they are less likely than WT EVs to provoke an immunogenic response *in vivo* and are unlikely to lead to protection from *Candida* infections when administered prophylactically. This idea could form the basis of further study both *in vitro* and *in vivo*.

## 5 SUMMARY

Sur7, a component of the MCC complex is enriched in EVs from WT *Candida* cells. Here we investigated the effect of a SUR7 gene knockout on the production and properties of EVs. The knockout of SUR7 leads to EVs with a reduced mean diameter as well as an altered protein and carbohydrate cargo. The EVs from *sur7Δ* contain transmembrane transporters which in WT are retained in the plasma membrane. Furthermore, proteins that protect fungal cells from human neutrophils together with proteins involved in pathogenesis were absent or diminished in the *sur7Δ* EVs compared to WT EVs. The reduced virulence of the *sur7Δ* cells in both *in vitro* macrophage killing assays and in *in vivo* insect models may result in part from EVs that lack pathogenesis related proteins that are either not produced by the cell or fail to be packaged into EVs. This is supported by the observation that virulence was partially restored with EVs isolated from a WT strain. Future work will focus on the interaction of the EVs from these lines with human cell lines to better understand how the structure and cargo of EVs is related to fungal pathogenesis. An understanding of the role that fungal EVs play in infection is expected to greatly facilitate the discovery of new drug targets and new therapies for the treatment of fungal diseases.

## AUTHOR CONTRIBUTIONS

**James A. McKenna**: Conceptualization; Formal analysis; Investigation; Methodology; Project administration; Writing—original draft; Writing—review & editing. **Donovan Garcia‐Ceron**: Formal analysis; Methodology; Writing—review & editing. **Mark R. Bleackley**: Methodology; Writing—review & editing. **Long Yu**: Formal analysis; Investigation; Writing—review & editing. **Vincent Bulone**: Formal analysis; Methodology; Writing—review & editing.**Marilyn A. Anderson**: Formal analysis; Funding acquisition; Project administration; Supervision; Writing—review & editing.

## CONFLICT OF INTEREST STATEMENT

The authors declare no conflicts of interest.

## Supporting information

Supporting Information

Supporting Information

Supporting Information

Supporting Information

Supporting Information
