# Supplementary figures and images for: SUR7 deletion in Candida albicans impacts extracellular vesicle features and delivery of virulence factors
Source: J Extracell Biol. 2023 May 2;2(5):e82. doi: 10.1002/jex2.82 (PMC11080841; doi:10.1002/jex2.82)

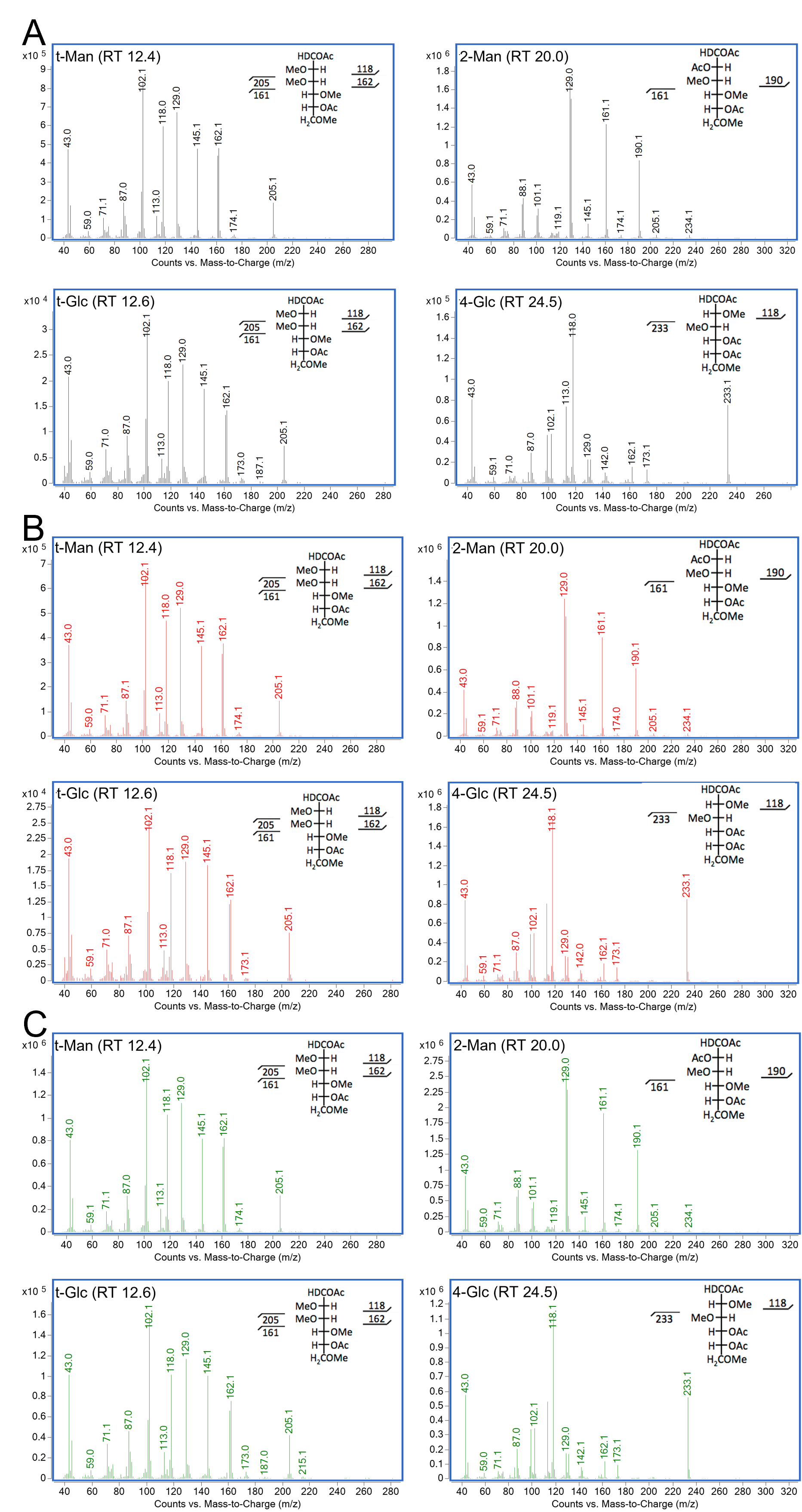

Supplement: Supplementary file 1 — Supporting Information [file JEX2-2-e82-s001.tif]
