# Supplementary material for: SUR7 deletion in Candida albicans impacts extracellular vesicle features and delivery of virulence factors
Source: J Extracell Biol. 2023 May 2;2(5):e82. doi: 10.1002/jex2.82 (PMC11080841; doi:10.1002/jex2.82)
Supplement: Supplementary file 2 — Supporting Information [file JEX2-2-e82-s003.docx]

Supplementary Table 1. P-values for all in vivo treatments compared to all other treatments

|  | **PBS-** | **WT** | **WT w *sur7Δ* EVs** | ***sur7Δ*** | ***sur7Δ* w WT EVs** | **PBS w WT EVs** |
| --- | --- | --- | --- | --- | --- | --- |
| **WT** | <0.0001 |  |  |  |  |  |
| **WT w *sur7Δ* EVs** | <0.0001 | 0.8468 |  |  |  |  |
| ***sur7Δ*** | 0.0047 | <0.0001 | <0.0001 |  |  |  |
| ***sur7Δ* w WT EVs** | <0.0001 | <0.0001 | <0.0001 | 0.0113 |  |  |
| **PBS w WT EVs** | 0.3751 | <0.0001 | <0.0001 | 0.3889 | 0.0028 |  |
| **PBS w *sur7Δ* EVs** | 0.9995 | 0.0005 | 0.0002 | 0.1125 | 0.0175 | 0.5586 |
