# Supplementary material for: SUR7 deletion in Candida albicans impacts extracellular vesicle features and delivery of virulence factors
Source: J Extracell Biol. 2023 May 2;2(5):e82. doi: 10.1002/jex2.82 (PMC11080841; doi:10.1002/jex2.82)
Supplement: Supplementary file 3 — Supporting Information [file JEX2-2-e82-s004.docx]

Supplementary Table 2. Expanded Table 2. Twenty most abundant proteins found in *sur7Δ* EVs but not WT EVs

| **Rank** | **Protein ID** | **description** |
| --- | --- | --- |
| 19 | HSP30 | Putative heat shock protein; fluconazole repressed; amphotericin B induced; Spider biofilm induced; rat catheter biofilm induced |
| 24 | C6_02330W | Described as a Gag-related protein; hyphal induced; downregulation correlates with clinical development of fluconazole resistance; repressed by nitric oxide, 17-beta-estradiol, ethynyl estradiol |
| 28 | CTR1 | Copper transporter; transcribed in low copper; induced Mac1, Tye7, macrophage interaction, alkaline pH via Rim101; 17-beta-estradiol repressed; complements S. cerevisiae ctr1 ctr3 copper transport mutant; flow model/Spider biofilm induced |
| 45 | GAP4 | High-affinity S-adenosylmethionine permease; required for SAM-induced morphogenesis; hyphal induced; regulated by Hap43, Gcn2 and Gcn4; colony morphology-related gene regulation by Ssnp |
| 48 | ENA21 | Predicted P-type ATPase sodium pump; Gcn4p-regulated; flucytosine, amphotericin B, or ketoconazole-induced; osmotic stress-induced; overlaps orf19.5170.1, which is annotated as a blocked reading frame; Spider biofilm induced |
| 82 | CAT1 | Catalase; resistance to oxidative stress, neutrophils, peroxide; role in virulence; regulated by iron, ciclopirox, fluconazole, carbon source, pH, Rim101, Ssn6, Hog1, Hap43, Sfu1, Sef1, farnesol, core stress response; Spider biofilm induced |
| 83 | FRE10 | Major cell-surface ferric reductase under low-iron conditions; 7 transmembrane regions and a secretion signal predicted; Tup1, Rim101, Ssn6, Hog1, caspofungin repressed; ciclopirox olamine induced; rat catheter biofilm induced |
| 104 | C4_02340W | Putative membrane protein; induced by alpha pheromone in SpiderM medium; Hap4-induced gene; Spider biofilm induced |
| 128 | FRP3 | Putative ammonium transporter; upregulated in the presence of human neutrophils; fluconazole-downregulated; repressed by nitric oxide; Spider biofilm induced; rat catheter biofilm repressed |
| 132 | SIT1 | Transporter of ferrichrome siderophores, not ferrioxamine B; required for human epithelial cell invasion in vitro, not for mouse systemic infection; regulated by iron, Sfu1, Rfg1, Tup1, Hap43; rat catheter and Spider biofilm induced |
| 133 | C1_08900W | Putative lipid raft associated protein; Spider biofilm induced |
| 142 | CR_08990C | Putative protein of unknown function; shows colony morphology-related gene regulation by Ssn6p |
| 144 | ZRT1 | Putative zinc transporter; acts with Pra1 in sequestration of zinc from host tissues during infection; hyphal, macrophage-induced; alkaline induced upon adherence to polystyrene; induced in oralpharyngeal candidasis; Spider biofilm induced |
| 152 | SLP3 | Plasma membrane protein implicated in stress response; similar to stomatin mechanoreception proteins; overexpression induces apoptotic-like cell death; absent from hyphal cells; induced by Rgt1; rat catheter and Spider biofilm induced |
| 169 | NCE102 | Non classical protein export protein; localized to plasma membrane; Hap43-induced gene; flow model biofilm induced; Spider biofilm induced |
| 202 | NHP6A | Putative non-histone chromatin component; RNA abundance regulated by tyrosol,cell density; Hap43-induced; amphotericin B, caspofungin repressed; 5'-UTR intron; rat catheter and Spider biofilm repressed |
| 207 | GLR1 | Glutathione reductase; upregulated by human neutrophils; oxidative stress-induced regulation via Cap1p; overexpression correlates with multidrug resistance in a cap1 mutant, farnesol induced; stationary phase enriched protein |
| 208 | C1_08900W | Protein of unknown function; hyphal-induced expression, regulated by Cyr1, Ras1, Efg1; Hap43-induced gene; Spider biofilm induced |
| 212 | CR_06500C | Protein of unknown function; Hap43-induced; regulated by Nrg1, Tup1; repressed by alpha pheromone in SpiderM medium; Spider biofilm induced; Bcr1-repressed in RPMI a/a biofilms |
| 215 | CR_06510W | Protein of unknown function; Hap43-induced; rat catheter and Spider biofilm induced |
