# Supplementary material for: SUR7 deletion in Candida albicans impacts extracellular vesicle features and delivery of virulence factors
Source: J Extracell Biol. 2023 May 2;2(5):e82. doi: 10.1002/jex2.82 (PMC11080841; doi:10.1002/jex2.82)
Supplement: Supplementary file 4 — Supporting Information [file JEX2-2-e82-s002.docx]

Supplementary Table 3. Expanded Table 3. Twenty most abundant unique proteins from WT EVs

| **Rank** | **Protein IDs** | **Description** |
| --- | --- | --- |
| 49 | ALS4 | GPI-anchored adhesin; role in adhesion, germ tube induction; growth, temperature regulated; expressed during infection of human buccal epithelial cells; repressed by vaginal contact; biofilm induced; repressed during chlamydospore formation |
| 58 | RBE1 | Pry family cell wall protein; Rim101, Efg1, Ssn6, alkaline repressed; O-glycosylation; no GPI anchor predicted; ketoconazol induced; regulated by Sef1, Sfu1, Hap4; flow model biofilm induced; rat catheter and Spider biofilm repressed |
| 78 | PLB4.5 | Phospholipase B; Hog1-induced; regulated by Ssn6; putative GPI-anchor; repressed during cell wall regeneration; clade-associated gene expression; Hap43-induced; rat catheter and Spider biofilm repressed |
| 89 | C2_10150W | Secreted protein; fluconazole-induced |
| 91 | KEX2 | Subtilisin-like protease (proprotein convertase); processes aspartyl proteinase Sap2; required for hyphal growth and wild-type virulence in mice; required for maturation of candidalysin Ece1p |
| 118 | CHT1 | Chitinase; putative N-terminal catalytic domain; has secretory signal sequence; lacks S/T region and N-glycosylation motifs of Chs2p and Chs3p; alkaline downregulated; expression not detected in yeast-form or hyphal cells |
| 134 | RBT1 | Cell wall protein with similarity to Hwp1; required for virulence; predicted glycosylation; fluconazole, Tup1 repressed; farnesol, alpha factor, serum, hyphal and alkaline induced; Rfg1, Rim101-regulated |
| 137 | ADH2 | Alcohol dehydrogenase; soluble in hyphae; expression regulated by white-opaque switching; regulated by Ssn6; indued by Mnl1 in weak acid stress; protein enriched in stationary phase yeast cultures; Spider biofilm induced |
| 148 | GDA1 | Golgi membrane GDPase, required for wild-type O-mannosylation, not N-glycosylation; required for wild-type hyphal induction, cell wall, and cell surface charge; not required for HeLa cell adherence; functional homolog of S. cerevisiae Gda1p |
| 158 | CR_10200W | Protein with a phosphoglycerate mutase family domain; Hap43-repressed gene |
| 178 | SAP7 | Pepstatin A-insensitive secreted aspartyl protease; self-processing; expressed in human oral infection; Ssn6p-regulated; role in murine intravenous infection; induced during, but not required for, murine vaginal infection; N-glycosylated |
| 206 | RBT4 | Pry family protein; required for virulence in mouse systemic/rabbit corneal infections; not filamentation; mRNA binds She3, is localized to hyphal tips; Hap43-induced; in both yeast and hyphal culture supernatants; Spider biofilm induced |
| 208 | SAP10 | Secreted aspartyl protease; roles in adhesion, virulence (RHE model), cell surface integrity; distinct specificity from Sap9; at cell membrane and wall; GPI-anchored; induced in low iron; Tbf1-activated; Spider biofilm induced |
| 225 | RPN2 | Putative 26S proteasome subunit; transcript regulated by Mig1; caspofungin repressed; regulated by Gcn2 and Gcn4; gene used for strain identification by multilocus sequence typing |
| 228 | DED1 | Predicted ATP-dependent RNA helicase; RNA strand annealing activity; Spider biofilm induced |
| 230 | C1_13260W | Ortholog(s) have FK506 binding, peptidyl-prolyl cis-trans isomerase activity and membrane localization |
| 234 | C4_06620C | Protein of unknown function; rat catheter and Spider biofilm induced |
| 237 | C2_10160W | Secreted protein; fluconazole-induced |
| 240 | LHS1 | Protein similar to S. cerevisiae Hsp70p; predicted Kex2p substrate; possibly essential, disruptants not obtained by UAU1 method; flow model biofilm repressed |
| 248 | PIR1 | 1,3-beta-glucan-linked cell wall protein; N-mannosylated, O-glycosylated by Pmt1; cell wall defect in het mutant; Hog1/fluconazole/hypoxia induced; iron/Efg1/Plc1/temp regulated; flow model biofilm induced; hyphal, Spider biofilm repressed |
